# The Satellite DNA PcH-Sat, Isolated and Characterized in the Limpet *Patella caerulea* (Mollusca, Gastropoda), Suggests the Origin from a Nin-SINE Transposable Element

**DOI:** 10.3390/genes15050541

**Published:** 2024-04-25

**Authors:** Agnese Petraccioli, Nicola Maio, Rosa Carotenuto, Gaetano Odierna, Fabio Maria Guarino

**Affiliations:** Department of Biology, University of Naples Federico II, Via Cinthia, I-80126 Naples, Italy; petra.ag@gmail.com (A.P.); nicola.maio@unina.it (N.M.); rosa.carotenuto@unina.it (R.C.); fabio.guarino@unina.it (F.M.G.)

**Keywords:** centromeric satellite DNA, satellite DNA origin, transposable elements, Nin-SINEM limpets

## Abstract

Satellite DNA (sat-DNA) was previously described as junk and selfish DNA in the cellular economy, without a clear functional role. However, during the last two decades, evidence has been accumulated about the roles of sat-DNA in different cellular functions and its probable involvement in tumorigenesis and adaptation to environmental changes. In molluscs, studies on sat-DNAs have been performed mainly on bivalve species, especially those of economic interest. Conversely, in Gastropoda (which includes about 80% of the currently described molluscs species), studies on sat-DNA have been largely neglected. In this study, we isolated and characterized a sat-DNA, here named PcH-sat, in the limpet *Patella caerulea* using the restriction enzyme method, particularly *Hae*III. Monomeric units of PcH-sat are 179 bp long, AT-rich (58.7%), and with an identity among monomers ranging from 91.6 to 99.8%. Southern blot showed that PcH-sat is conserved in *P. depressa* and *P. ulyssiponensis*, while a smeared signal of hybridization was present in the other three investigated limpets (*P. ferruginea*, *P. rustica* and *P. vulgata*). Dot blot showed that PcH-sat represents about 10% of the genome of *P. caerulea*, 5% of that of *P. depressa*, and 0.3% of that of *P. ulyssiponensis*. FISH showed that PcH-sat was mainly localized on pericentromeric regions of chromosome pairs 2 and 4–7 of *P. caerulea* (2n = 18). A database search showed that PcH-sat contains a large segment (of 118 bp) showing high identity with a homologous trait of the Nin-SINE transposable element (TE) of the patellogastropod *Lottia gigantea*, supporting the hypothesis that TEs are involved in the rising and tandemization processes of sat-DNAs.

## 1. Introduction

Limpets of the genus *Patella* Rafinesque, 1815 are popular marine gastropod grazers. The genus includes 16 species living on rocky intertidal areas of the East Atlantic and Mediterranean coasts [1].

Five species of *Patella* inhabit the Mediterranean Sea. Other than the historically documented presence of four species, *P. caerulea* Linnaeus, 1758, *P. ferruginea* Gmelin, 1791, *P. rustica* Linnaeus, 1758, and *P. ulyssiponensis* Gmelin, 1791, [2,3], an additional species, *P. depressa* Pennant, 1777, has been recently recorded in western Mediterranean coasts [4]. The above first four *Patella* species can be sympatric on intertidal rocky areas, but distributed at different vertical levels, with *P. rustica* at the supralittoral zone; *P. ferruginea* at the upper mesolittoral level; *P. ulyssiponensis* at the lower mesolittoral; and *P. caerulea* in both the intertidal and upper infralittoral zones [3,4]. 

*Patella ferruginea* deserves particular attention because the species is threated and in “danger of extinction” according to the Barcelona Convention [5] and it is the most endangered marine species on the list of the European Council Directive 92/43/EEC on the Conservation of Natural Habitat of Wild Fauna and Flora [6]. The other three limpet species, *P. caerulea*, *P. rustica*, and *P. ulyssiponensis*, are quite common in the Mediterranean Sea, with *P. caerulea* locally abundant, and used for food, fishery, and as a species index in marine environmental monitoring studies (e.g., [6,7,8,9]). 

In accordance with their ecological and commercial importance in marine ecosystems, interest in genetic and molecular research on *Patella* has increased in recent years [10,11,12,13,14,15,16]. Recent genome assemblies are available on online databases (direct submission by the Wellcome Sanger Tree of Life Programme [17,18]). However, cytogenetic aspects are largely unexplored, and of 16 species of the genus [1], only 3 (*P. caerulea*, *P. rustica*, and *P. ulyssiponensis*) have been karyotyped. *Patella caerulea* and *P. rustica* have 2n = 18 chromosomes (with seven biarmed pairs, 1–7, and two telocentric pairs, 8–9) while *P. ulyssiponensis* has 2n = 16 elements (with all biarmed pairs) [2,19]. Furthermore, heterochromatin is mostly located on the pericentromeric regions and presents a complex composition [2], which notoriously is mostly constituted by repeated, non-coding DNA sequences, particularly satellite DNA (sat-DNA) [20,21]. Sat-DNA constitutes a large component of eukaryotic genomes, frequently localized on centromeric and telomeric regions of chromosomes [22,23]; it is constituted of arrays of tandemly repeated sequences, with monomeric units ranging from lower than 100 to more than 1000 bp and copy numbers varying from lower than a few hundred to several million copies and reaching more than 50% of the genome [24,25,26,27].

Sat-DNA was previously considered as junk and selfish DNA [28,29]. However, in the last two decades, evidence has been accumulated showing that sat-DNA has substantial roles in contributing to the assembly of centromeric chromatin, chromosome segregation and gametogenesis, higher-level organization of the nucleus, reproductive isolation and speciation, and the architecture and integrity of the genome (references in [23]). Furthermore, changes in the copy number and rates of expression of sat-DNAs have also been proposed be involved in stress, tumorigenesis, and adaptation to environmental changes (references in [23]).

The high similarity of repetitive sequences of sat-DNAs makes it difficult to reconstruct the sequential order of the monomeric units. Study problems also arise because sat-DNAs are under-represented and/or commonly missed in the annotated assemblies deposited in public repositories [30,31,32,33,34]. These difficulties have started to be overcome only recently, thanks to the development of powerful bioinformatic programs and high-quality annotated assemblies, which, so far, have been applied mostly to humans and model species [18,27,30,32,33,35,36,37].

In non-model species, such as molluscs, previous and relatively old methods have been and can still be useful tools to study sat-DNAs. For example, gradient density methods were used to first isolate sat-DNAs. In fact, sat-DNAs appear as a satellite of the main bulk of the DNA peak, from which the name sat-DNA was originally conceived [21,37]. A successive and more widely applied method is represented by restriction enzymes [38]. In the present paper, the digestion of the genomic DNA of the limpet *P. caerulea* with the restrictase *Hae*III was used to evidence the presence of a sat-DNA, here named PcH-sat, whose monomeric units were isolated and sequenced. The chromosomal distribution of PcH-sat and its quantification in the genome of *P. caerulea* were assayed by FISH and dot blot, respectively. Finally, the conservation of PcH-sat in Patellogastropoda and other mollusc taxa was assayed by Southern blot and queried in annotated assemblies.

## 2. Material and Methods

Experimental procedures were conducted on the following species of the genus *Patella*: *P. caerulea*, *P depressa*, *P. ferruginea*, *P. rustica*, *P. ulyssiponensis*, and *P. vulgata*. The number and provenance of the studied *Patella* specimens are given in Table 1.

Several samples considered here have been already used in other studies [2,38,39]. 

### 2.1. DNA Extraction

DNA was extracted from the foot of the studied specimens according to Sokolov [40]. In brief, a piece of foot (4–5 mm), finely cut with forceps, was transferred in a 2 mL plastic tube containing 1 mL of the lysis buffer (50 mM Tris-HCl, pH 7.5, 100 mM NaCl, 10 mM EDTA, 1% sodium dodecyl sulphate (SDS), 0.2 mg/mL Proteinase K) and incubated at 55 °C until complete digestion. A saturated solution of KCl (100 μL) was added and samples were incubated on ice for 5 min. After centrifugation at 12,000 rpm, the supernatant was treated twice with an equal volume of a chloroform/isoamyl alcohol (24:1) mixture. DNA was extracted with 100% ethanol, washed in 70% ethanol, centrifugated a 6000 rpm, air-dried, and finally dissolved in an adequate volume of TE buffer (10 mM Tris-HCl, 1 mM EDTA). 

Preliminary digestions of the DNA of *P. caerulea* with several restriction enzymes, *Bgl*II, *Eco* RI, *Hae*III, *Hpa*II, *Msp*I, and *Taq*I, evidenced a ladder of bands in the DNA digested with *Hae*III. After that, 5 μg DNA of *P. caerulea* was digested overnight with 10 units of *Hae*III (Promega, Madison, WI, USA). The monomeric unit of about 180 bp was eluted by the Qiaquick gel extraction kit (Qiagen, Hilden, Germany) and ligated in pGem-T easy vector (Promega, Madison, WI, USA). 

After the transformation of monomeric units in DH5α cells, positive colonies were selected and amplified by PCR using primer pairs T7 (5′ TAATACGACTCACTATAGGG 3′) and SP6 (5′ ATTTAGGTGACACTATAG 3′) with the following PCR conditions: 5 min at 94 °C; 36 cycles at 94 °C for 30 s, 50 °C for 30 s, and 70 °C for 45 s; and 5 min at 72 °C. Sequencing of positive colonies (about 50) was performed in both orientations using the BigDye Terminator kit v1.1 Cycle Sequencing Kit (Thermo Fisher, Waltham, MA, USA) and the automatic sequencer ABI Prism 310 (Applied Biosystems, Foster City, CA, USA). Sequences presenting similar traits were selected and used to design the following primer pair: PcH-sat F 5′ ACCGCCGCTKCCCCCCTAA 3′ and PcH-sat R 5′ TATAATAAATAAGCAACATAGAGAAAA 3′. These were used to amplify the isolated sat-DNA from the genomic DNA of *P. cerulean*, *P. ulyssiponensis*, *P. ferruginea*, *P. rustica*, *P. depressa*, *P. intermedia*, and *P. vulgata*. The PCR conditions with the primer pair PcH-sat F and PcH-sat R were as follows: 5 min at 94 °C; 36 cycles at 94 °C for 30 s, 65 °C for 30 s, and 70 °C for 45 s; and 5 min at 72 °C. Amplicons were purified and sequenced in both directions using the same primer pair.

Clone 2 of PcH-sat of *P. caerulea* was biotinylated and used for Southern blot, quantitative dot blot, and FISH analyses (see below).

### 2.2. Southern Blot

Southern blots were performed according to [41] and carried out on 5 μg of *P. caerulea* DNA digested with 10 units of *Hae*III at 0, 5, and 15 min; 1 h; and overnight.

For the other investigated molluscan species (*P. depressa*, *P. vulgata*, *P. rustica*, *P. ferruginea*, *P. ulyssiponensis*), Southern blot was performed on 5 μg of DNA digested overnight with *Hae*III. Hybridization was carried out as described by [41] using the biotinylated clone 2 of PcH-sat. Washing was performed at medium stringency conditions [2] washes in 0.2× standard saline citrate solution (SSC) and 0.5% sodium dodecyl sulphate (SDS) for 15 min. at 55 °C. Hybridization signals were revealed by 5-bromo-4-chloro-3-indolyl phosphate and nitro blue tetrazolium (BCIP-NBT) (Promega, Madison, WI, USA).

### 2.3. Quantitative Dot Blot

For quantitative dot blot analysis, 1 μg/mL of DNA of *P. caerulea*, *P. depressa*, *P. vulgata*, *P. rustica*, *P. ferruginea*, and *P. ulyssiponensis* was added to denaturing buffer (0.4 M NaOH, 1 M NaCl), and serially diluted six times with an equal volume of *E. coli* DNA at a concentration of 1 μg/mL. A purified sample of PcH-sat amplicons of *P. caerulea* was used as a standard at an initial concentration of 0.1 μg/mL + 0.9 μg/mL of *E. coli* DNA in denaturing buffer. A 100 μL aliquot of each dilution was added per slot in a dot blot apparatus (Bio-Rad, Hercules, CA, USA), filtered on a nylon membrane (Sigma-Aldrich, St. Louis and Burlington, MA, USA), and fixed on the membrane by exposition for 4 min under transilluminator UV light. Hybridization and staining procedures were the same as those used for the Southern blot.

### 2.4. Genome Size Estimation

Genome size (GS) was determined for *P. caerulea* because it was only for this species that we had fresh tissues which were suitable for performing a Feulgen densitometric image analysis (FIA), according to [42]. The integrated optical densities (IODs) were converted to genome size (GS) in picograms/nucleus (pg/N) using the IODs of three standards of known GS: *Pecten maximus* (Linnaeus, 1758) (1.42 pg/N), *Podarcis siculus* Rafinesque, 1810, (2.20 pg/N), and *Bufotes viridis* (Laurenti, 1768) (4.83 pg/N) [43].

### 2.5. Fluorescence In Situ Hybridization (FISH)

Chromosomes were obtained from gonads as described in [44]. FISH staining was performed according to Petraccioli et al. [2]. Chromosomes were aged for 1 day at room temperature, then left for 2 h at 60 °C and incubated for 30 min in RNase at 100 μg/mL in Tris-HCl 10 mM at pH 6.5. After dehydration in alcohol series, the chromosomes and probe were denatured for 3 min at 72 °C in hybridization mixture (10 ng/mL biotinylated 16 dUTP probe + 0.1 mg/mL shared *E*. *coli* DNA in 2 × SSC with 50% formamide). Hybridization was carried out at 40 °C for 20 h, which was followed by washing in 1 × SSC at 72 °C for 5 min and at RT for 2 min. Probe detection was performed by chromosome incubation for one hour with monoclonal anti-biotin (Sigma cod. B7653) diluted 1:500 in PTB (1 mL PTB = 5 μL of Tween 20% + 0.01 g of dry milk in 1 mL of PBS 0.2 M), followed by washing in 1 × PBS and incubation for 30 min with FITC-conjugated anti-anti-biotin antibodies (Sigma) diluted 1:50 in PTB. After washing in 1 × PBS, chromosomes were counterstained with 5 μg/mL propidium iodide (PI) in 1 × PBS for 15 min at RT and mounted with antifade (DABCO, Sigma). The hybridization signals were detected and recorded using an epifluorescent microscope (Leica DM) equipped with a digital camera.

### 2.6. Bioinformatic Analysis

To test for the presence of PcH-sat or its traits in bioinformatic databases, queries were made to Repbase [45], Repeatmasker [46], and GenBank, with different BLAST suites and parameters [47]. In particular, we used blastn (nucleotide collection n/r; Reference RNA Sequences, Refseq_RNA; whole-genome shotgun contigs, WGS) and blastx (non-redundant protein sequences, n/r). In the BLAST search, queries were performed by setting filters for cover and identity > 70%.

## 3. Results

### 3.1. Restriction Enzyme and Sequence Analysis

**Figure 1:**
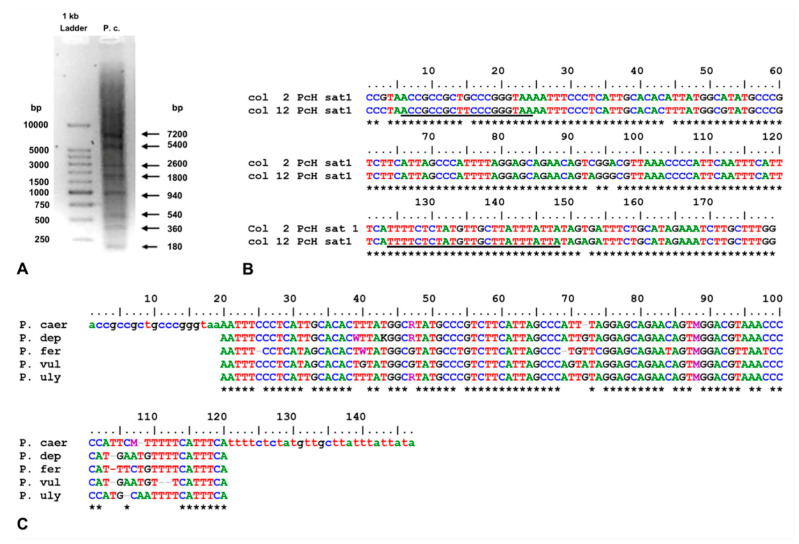
(**A**) A 1.5% agarose gel loaded with 5 μg of DNA of *P. caerulea* digested with *Hae*III; (**B**) Alignment of two clone sequences of monomeric unit of PcH-sat (underlined segments are the primer pair sequences used to amplify PcH-sat from genomic DNA of studied *Patella* species and the relative alignment of amplified sequences reported in (**C**); lower case = primer pair). * = base identity among aligned sequences.

DNA digestion of *P. caerulea* with *Hae*III restrictase evidenced a ladder of bands with a monomeric unit of about 180 bp, here named PcH-sat (Figure 1A). After the transformation of DH5α cells, clones displaying sequences with a length close to 180 bp and an identity among them >90% were selected (see Figure 1B) and used to design a primer pair to perform direct PCR amplification from the DNA of the studied *Patella* species. PcH-sat units were 179 bp long, AT-rich (58.7%), and with an identity among monomer units ranging from 91.6% to 99.8% (GenBank accession numbers PP554448–PP554449).

Successful amplifications were obtained in *P. caerulea*, *P. depressa*, *P. ferruginea*, *P. ulyssiponensis*, and *P. vulgata*. The alignment of the obtained sequences against *P. caerulea* showed nearly 100% coverage and an identity ranging from 89.1% (*P. ferruginea*) to 96.5% (*P. ulyssiponensis*), with variations mostly in the 3′ tail of the sequences (Figure 1). Direct PCR amplification was unsuccessful for the DNA of *P. rustica* and of several marine and air breath gastropod species belonging to different genera and families (evidence not shown). However, Southern blot and dot blot revealed the occurrence of hybridization signals in *P. rustica*. Additional primer pairs are probably needed for direct PCR amplification of PcH-sat in this limpet species.

### 3.2. Southern Blot

**Figure 2:**
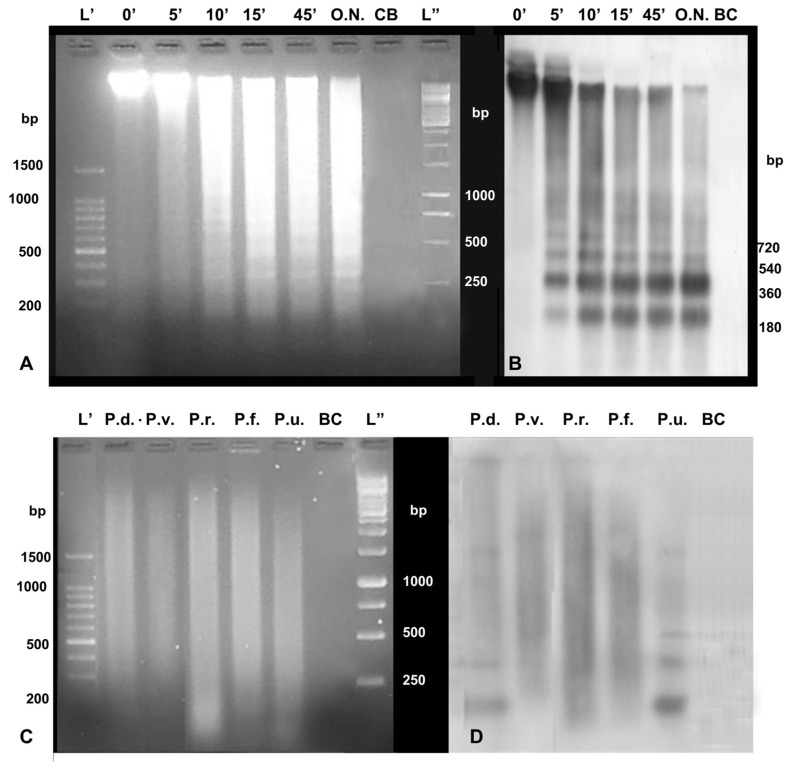
A 1.5% agarose gel (**A**,**C**) loaded with 5 µg of DNA of *P. caerulea* quickly digested with *Hae*III (**A**), and 5 µg of DNA of *P. depressa* (P.d.), *P. vulgata* (P.v,), *P. rustica* (P.r.), *P. ferruginea* (P.f.), and *P. ulyssiponensis* (P.u.) digested O.N. with *Hae*III (**C**), and the relative Southern blots probed with biotinylated genomic PcH-sat amplicons of *P. caerulea* (**B**,**D**) (BC = Blank Control).

Southern blot with the DNA of *P. caerulea* digested with *Hae*III confirmed the DNA satellite profile of PcH-sat and the length of its monomeric unit (179 bp), also evidencing that hybridization signals were more abundant on the dimeric band than the monomeric one (Figure 2A,B). A smeared hybridization signal was also present when the DNA was exhaustively digested (O.N.) (Figure 2A,B).

The Southern blot results for the overnight-digested DNA of other studied *Patella* species evidenced a ladder of bands with a monomeric band of about 180 bp in *P. depressa* and *P. ulyssiponensis*, with hybridization signals mostly on the monomeric unit (Figure 2C,D). In *P. ferruginea*, *P. vulgata*, and *P. rustica*, only a smeared hybridization signal was observed. 

### 3.3. Quantitative Dot Blot

In the dot blot analysis, we compared the density of the dilutions of the genomic hybridization of each studied *Patella* species against scalar quantities of the clone of PcH-sat (100%, 50%, 25%, 12.5%, 6.25%, and 0.32% of the PcH-sat clone). To calculate the genomic quantity (in %) of PcH-sat in the genome of the study samples, the selected clone percentage was divided by 10, because the quantity of the genomic DNA is 10 times (1 mcg) higher of that of the clone (0.1 mcg).

**Figure 3:**
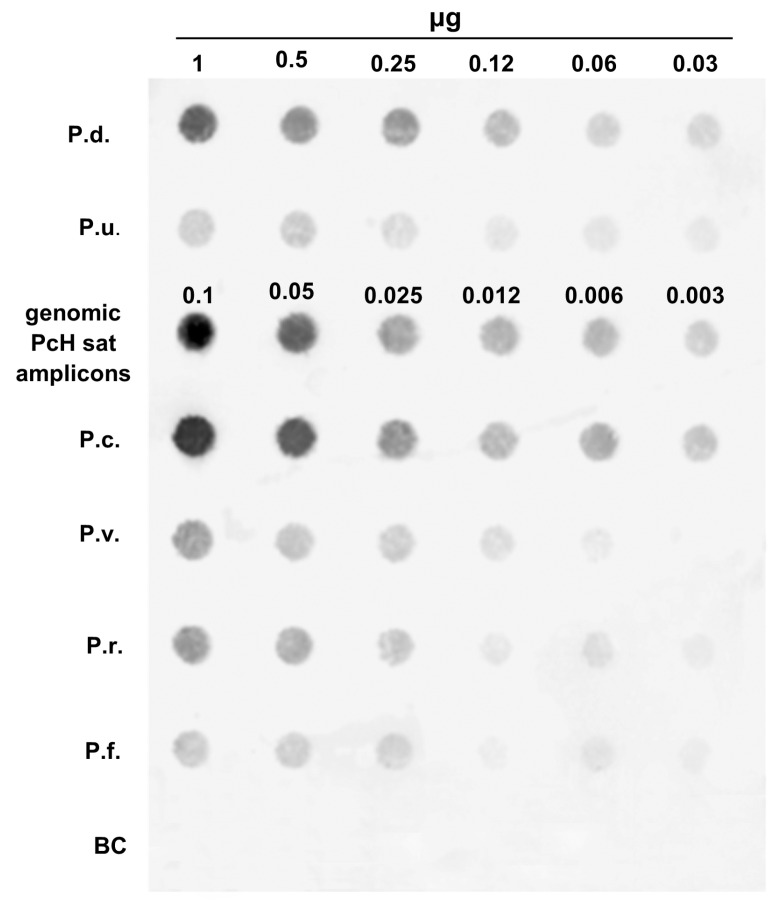
Quantitative dot blot on scalar DNA amounts (10, 5, 2.5, 1.25, 1, and 0.5 μg) of *P. depressa* (P.d.), *P. ulyssiponensis* (P.u.), *P. caerulea*, *P. vulgata* (P.v.), *P. rustica* (P.r.), and *P. ferruginea* (P.f.) and scalar amounts (1.0, 0.5, 0.25, 0.125, 0.01, and 0.005 μg) of genomic amplicons of *P. caerulea* probed with the biotinylated clone 2 of PcH-sat of *P. caerulea* (BC = Blank Control).

Based on the quantitative dot blot analyses (Figure 3), PcH-sat accounts for about 10% of the genome of *P. caerulea*, 5% of *P. depressa*, 2% of *P. rustica*, 1.2% of *P. vulgata*, 0.3% of *P. ulyssiponensis*, and less than 0.3% of *P. ferruginea*. The copy number of PcH-sat was evaluated on the genome size (GS) of *P. caerulea*, which has been determined in this work as GS = 1.09 pg/N, and *P. depressa*, whose GS ranges from 0.95 to 1.46 pg/N as evaluated by [48] in their draft genome assembly analysis on the species. Therefore, PcH-sat is present with about 600,000 copies in the genome of *P. caerulea*, and with about 60,000–90,000 copies in *P. depressa* (according to the minimum and maximum GS values estimated by [48].

### 3.4. FISH

**Figure 4:**
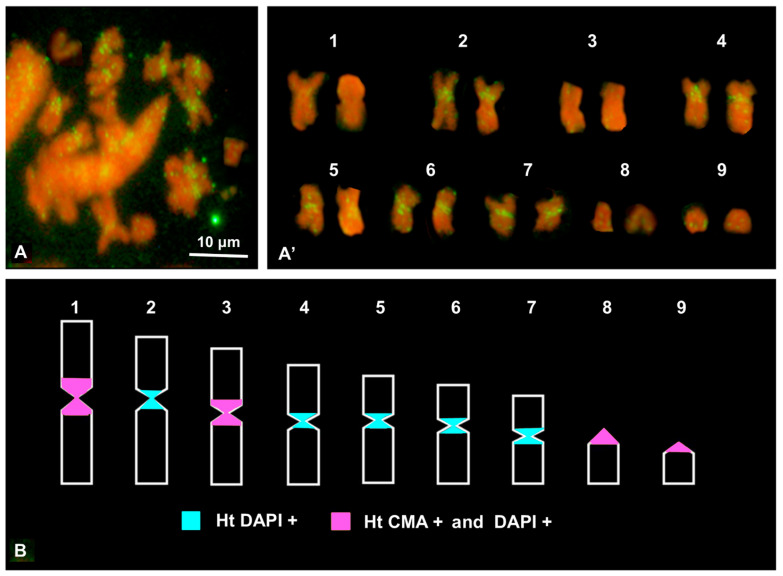
Metaphase plate (**A**) and the relative karyotype (**A′**) probed with biotinylated genomic PcH-sat amplicons of *P. caerulea*. (**B**) Haploid ideogram of the C-banding +CMA + DAPI pattern, here originally redrawn from [2].

Metaphase chromosome plates, suitable for FISH, were available only from the gonads of *P. caerulea*. There were abundant hybridization signals on the pericentromeric regions of metacentric pairs 2 and 4–7, which then colocalized with the distribution of DAPI + heterochromatin [2] (Figure 4A,B). In addition, interspersed signals were observed along both arms of all chromosomes (Figure 4A,A′).

### 3.5. Repeatmasker and Repbase Queries

The search in Repeatmasker did not produce hits, evidencing the lack of simple repeats in PcH-sat.

**Figure 5:**
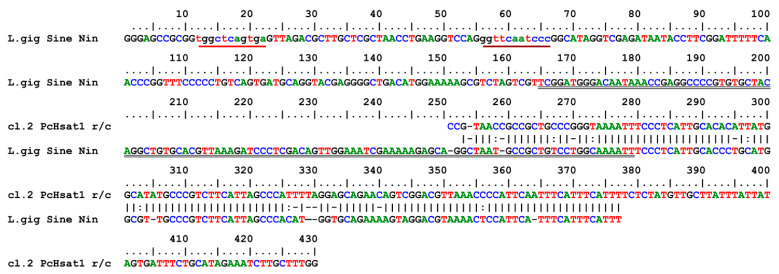
Alignment between the consensus sequence of Lgi-Nin-DC-SINE1 of *L. gigantea* [49] and the sequence of clone 2 of *P. caerulea* PcH-sat. Red and brown underlined lower-case traits refer to Box A and B for DNA polIII, respectively. The double underlined segment refers to Nin domain of *L. gigantea*. Colons (:) show transitions.

The query in the Giri Repbase database evidenced a trait of 118 bp (from 8 to 127) of PcH-sat, showing an identity of 84.6% (score 650; direction +/+) with a homologous segment of the Non-LTR Retrotransposon SINE2/tRNA Nin-SINE of the patellogastropod *Lottia gigantea* G. B. Sowerby I, 1834 [49]. In Figure 5, we show the alignment between clone 2 of PcH-sat of *P. caerulea* and the whole Nin-Sine sequence of *L gigantea* (369 bp long, with evidence of Box A and Box B for DNA polymerase *Pol*III and the Nin domain). PcH-sat contains a large 3′ truncation of the Nin-SINE of *L. gigantea* (from 253 to 377 bp; identity 90.3%). Furthermore, only a short 5′ segment of PcH-sat (26 bp) shows identity (83.7%) with the Nin domain of the Nin-SINE of *L. gigantea* [49] (Figure 5).

### 3.6. BLAST Analysis

**Figure 6:**
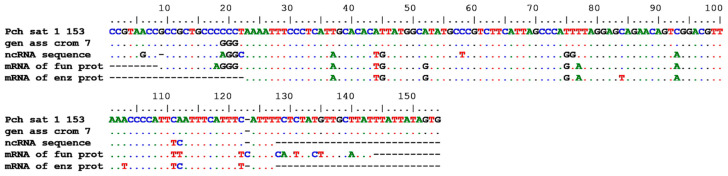
Alignment among the PcH-sat (from 1 to 153) and homologous traits, from up to down, of the chromosome 7 genome sequence of *P. depressa* (AN OX419721), an uncharacterized ncRNA of *P. vulgata* (AN XR_007683079), a nuclear hormone receptor mRNA of *P. vulgata* (AN XM_050542854), and an ATP-dependent RNA helicase mRNA of *P. vulgata*.

The query to n/r collections with filters at 70% identity and 70% cover produced 83 hits: 27 of these were for the genome assembly sequences of chromosomes 1–9 of *P. depressa*, *P. pellucida*, and *P. vulgata*; 55 hits were for the nuclear RNAs and transcript mRNAs of structural and functional proteins of *P. vulgata*; and 1 hit was for a microsatellite region of *P. ferruginea*. All the hits covered the region from 1 to 153 of PcH-sat. In Figure 6 is shown the alignment among segments 1–153 of PcH-sat, a homologous region of the genome assemblies of *P. depressa* chromosome 7, and the ncRNAs and structural and functional mRNAs of *P. vulgata*.

**Figure 7:**
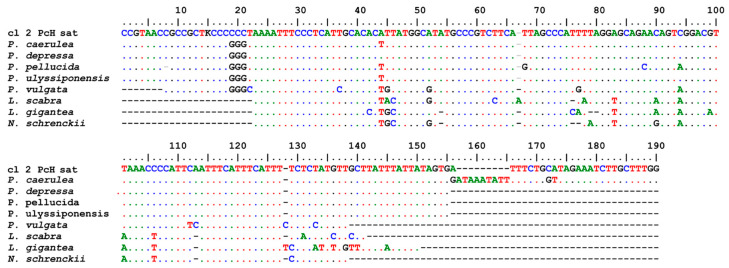
The alignment of clone 2 PcH-sat sequence of *P. caerulea* vs. the homologous trait of the first hits of deposited WGS archives of *Patella*, *Lottia*, and *N. schrenkii* species.

The search in the WGS of *Patella* (taxid 6463), with filters set for cover and identity > 70%, produced 2318 hits, of which 29 were for *P. caerulea*, 357 were for *P. depressa*, 720 were for *P. pellucida*, 557 were for *P. ulyssiponensis*, and 669 were for *P. vulgata* ([50], direct submission by the Wellcome Sanger Tree of Life Programme). The query extended in Mollusca only retrieved hits in the deposited WGS Patellogastropoda assemblies, namely of the Lottidae family (*L. scabra* (Gould, 1846), 111 hits; *L. gigantea*, 113; and *Nipponacmea schrenkii* (Lischke, 1868)) 16 hits ([17]; Dawson et al., direct submission; Qu. and Wang, direct submission) (see Table 2). The alignment of the PcH-sat sequence of *P. caerulea* vs. the homologous trait of the first hits of the deposited WGS archives of *Patella*, *Lottia*, and *N. schrenkii* species is shown in Figure 7.

The query with the PcH-sat of *P. caerulea* to Blastx did not produce hits.

## 4. Discussion

Studies of sat-DNAs in molluscs are fragmentary and relative to a small number of species, mostly bivalves, because of their economic importance and relevant roles in marine environments (reviewed by [51]). This kind of analysis in gastropods is rarely performed and only one study is reported in the literature, with gradient density centrifugation methods applied to the DNA of the muricid *Rapana venosa* (Valenciennes, 1846), formerly *R. thomasiana* Crosse, 1861 [52]. The isolated sat-DNA in the murid species was about 5% of the genome, AT-rich, and with monomeric units of 1460 bp, which probably originated from an ancestral 400–500 bp long sequence [52]. The sat-DNA isolated here from *Patella carulea*, named PcH-sat, only shares a relatively AT-rich content (about 59%), while its monomeric units are much shorter in length than the sat-DNA of *R. venosa* and its supposed ancestral sequence. 

We are inclined to classify PcH-sat as a centromeric sat-DNA, based on its monomeric length (179 bp), high AT content, and the presence of regions of five or six adenines/thymines repeated in phase. These sat-DNAs mostly localize in centromeric or pericentromeric heterochromatin, and the presence of blocks of five or six adenines repeated in phase have been frequently reported to be involved in heterochromatin condensation [31,38,53,54]. Finally, FISH results definitively localize PcH-sat in the centromeric/pericentromeric regions of five out nine chromosomes of *P. caerulea* (see Figure 4). The interspersed hybridization signals were probably due to the truncated segment of the Nin-SINE elements of *L. gigantea* occurring in all chromosomes (see also below). This situation mirrors that of Cg170/HindIII satDNA of *Crassostrea gigas*, where FISH analyses showed its localization in centromeric regions of several chromosome pairs as well as interspersed signals on all chromosomes [55]. Interestingly, Tunjić Cvitanić et al. [33,34] recently proposed that signal interspersion was related to the activity of TEs, in particular of Helitrons.

The Southern blot analysis, besides the tandem organization characteristic of sat-DNAs, showed that PcH-sat is mostly present in monomeric or dimeric units in *P. caerulea*, *P. depressa*, and *P. ulyssiponensis*, suggesting a strong conservation of the *Hae*III restriction site and that events of deletion/insertion seem to be excluded and or minimized in the monomers in the PcH-sat of the three scallop species.

The dot blot results evidence that PcH-sat is present in the genomes of *P. caerulea*, *P. depressa*, and *P. ulyssiponensis*, respectively, for about 10%, 1.2%, and 0.3%. Based on the genome size of GS = 1.09 pg/N, here determined for the former species, and the GS = 0.95–1.46 for *P. depressa*, as evaluated by [48], the copy numbers were 600,000 in *P. caerulea* and 60,000–90,000 in *P. depressa*. The amounts resulting from the relative signals interspersed along the chromosomes must be deducted from these percentages. However, the quantitative percentages of PcH-sat in *P. depressa* and *P. ulyssiponensis* are in accordance with those revealed in bivalve mollucs, while Pch-sat in *P. caerulea* can be considered an abundant sat-DNA, lower only than the genome percentage (20%) of PjHhaI sat-DNA isolated by [39] in the scallops *Pecten jacobaeus* and *P. maximus* [51]. In addition to multimeric bands, smeared hybridization signals were also evidenced in *P. caerulea*, *P. depressa*, *P. ulyssiponensis*, *P. ferruginea*, *P. rustica*, and *P. vulgata*. The smeared signals are not due to PcH-sat, but to its segment showing high identity with the homologous trait of Nin-SINE of *L. gigantea* [49]. The latter is also responsible for the direct PCR amplicons obtained here in study limpet species, because the primer pair used includes the Nin-SINE segment. Similarly, this TE is also responsible for the positive hits obtained from the BLAST queries in the WGS of deposited assemblies of Archaegastropoda species (see Table 2). The differences between the number of PcH-sat sequences reported in WGS (see Table 2) and the quantification obtained here with the dot blot analysis in *P. caerulea*, *P. depressa*, and *P. ulyssiponensis* are probably due to the omission of repeated sequences in the WGS assemblies [30,31,32,33].

Nin-SINE- in *L. gigantea* was first studied by Piskurek and Jackson [49], who exhaustively described the conservation and evolution in Metazoans of the Nin domain. This TE penetrated the genome of the common ancestor of *Patella*, and vertical inheritance and its transposition activity deeply modified the genome of this genus of Patellogastropoda. The queries in Refseq-RNA and n/r sequence collection evidenced that truncated Nin-SINE copies of *L. gigantea* are transcribed, but not translated, resulting in their presence in the mRNAs of both structural and functional proteins of *P. depressa*, *P. pellucida*, and *P. vulgata* (see Figure 6). It should be noted that a significant role in the variability and evolution of the concerned genome has also been documented for other kinds of SINE elements. For example, RUDI SINE and SINE-Squam1 deeply affected the genome of molluscs and squamates, respectively [56,57,58].

In our case, the truncated segment of Nin-SINE of *L. gigantea* in PcH-sat is a non-active element because it lacks Box A and Box B for *pol*III. However, the occurrence and amplification of PcH-sat is clearly linked to Nin-SINE, supporting the hypothesis that the origin of sat-DNAs is mediated by TEs [23,38,59]. 

In fact, there is increasing evidence that TEs facilitate the dispersion of sat-DNA repeats and their tandem arrangement (tandemization), and different hypotheses have been proposed [59,60]. One of these involves Helitrons, which are able to incorporate variable numbers of tandem repeats and mediate their dispersion and amplification by rolling-circle replication events. In contrast, a different hypothesis reports the capture of sat-DNA sequences by a TE, while its following transposition cycles increase the genomic occurrence of monomeric units and tandem repeats, finally producing the classically observed repeated structure of sat-DNAs (for details see [23]). 

Whatever the mechanism involved is, it could be hypothesized that in *Patella* the tandemization of Nin-SINE, like *L. gigantea* sequences, and the origin of PcH-sat occurred in the common ancestor of this genus of Patellogastropoda with its successive conservation (in *P. caerulea*, *P. depressa*, and *P. ulyssiponensis*) or loss (as in *P. ferruginea*, *P. vulgata*, and *P. rustica)*. Alternatively, based on the phylogenetic relationships of the genus *Patella* by Sá-Pinto et al. [11], the studied species of *Patella* are in different clades, namely, (((*P.caerulea*, *P. depressa*) ((*P. ulyssiponensis*, *P. vulgata*) (*P. rustica*, *P. ferruginea*))), suggesting that the tandemization and growth of PcH-sat originated independently in the different species. The events of tandemization producing a genomic augmentation of PcH-sat were dependent on specific genomic properties allowing the tandemization process. Interestingly, the FISH results in *P. caerulea* also suggest that the events of tandemization, rise, and expansion in PcH-sat can occur only on specific chromosome regions. In fact, PcH-sat is only present on the pericentromeric regions of chromosomes 2 and 4–7, incidentally overlapping with heterochromatin positive to DAPI, and not on the pericentromeric regions of other chromosomes, which contain a heterochromatin positive to Chromomycin A_3_ and DAPI [2]. Interestingly, in other chromosomal regions of *P. caerulea* the process of tandemization of PcH-sat is hampered and this correlates with the very scarce presence of Nin-SINE of *L. gigantea* segments (some tens based on the query in WGS assembly with filters at 70% cover and identity). The low copy number of truncated Nin-SINE probably is a consequence of the ‘purifying selection’ process against TEs [60,61,62]. This process of purification is very effective in chromosomal regions other than those in the pericentromeric areas of chromosomes 2 and 4–7. In these pericentromeric regions, TE taming [63,64] appears loose and probably allowed the tandemization of PcH-sat. The processes of TE taming and tandemization also appear to vary in the different species. In fact, compared to *P. caerulea*, the copy number of PcH-sat appears to be much lower in *P. depressa* and *P. ulyssiponensis*, while the number of truncated Nin-SINEs is much higher (see Table 2).

Further bioinformatic, molecular, and cytogenetic analyses should be performed in order to provide an adequate description of the satellitome in the genus *Patella* as well as in other mollusc taxa.

## Figures and Tables

**Table 1:** Number and provenance of the studied samples of *Patella*.

Species	Locality	Number
*P. caerulea*	Pozzuoli, Naples, Italy	3
	Gaeta, Latina, Italy	2
*P. rustica*	Nisida, Naples, Italy	2
	Acciaroli, Salerno, Italy	1
*P. ulyssiponensis*	Gaiola, Naples, Italy	2
*P. vulgata*	Baiona, Spain	2
	Porto, Portugal	1
*P. depressa*	Porto, Portugal	2
*P. ferruginea*	Porticcio, Corse, France	1

**Table 2:** Results of query with clone 2 sequence of PcH-sat to BLAST WGS Patellogastropoda archives, filtered with 70% identity and 70% cover. Below: the alignments of the relative sequences of first hits against the sequence of clone 2 of PcH-sat of *P. caerulea*.

Species	Nr. Hits		Max Score	% Cover	E Value	% Identity	Orient.	Accession
*P. caerulea*	29	1st hit	285	98%	3.00 × 10^−75^	94129	+/−	JAZGQO010000021
		Last hits	233	93%	2.00 × 10^−59^	93.092	+/−	JAZGQO010000031
*P. depressa*	340	1st hit	260	100%	3.00 × 10^−68^	97.39%	+/+	CAOLEZ010000010
		Last hit	81.5	72%	6.00 × 10^−14^	76.58%	+/+	CAOLEW010000395
*P. pellucida*	726	1st hit	233	99%	1.00 × 10^−59^	94.08%	+/−	CAKJPO010001416 CAKJPO010001283
		Last hit	59.9	70%	2.00 × 10^−7^	73.08%	+/−	
*P. ulyssiponensis*	557	1st hit	279	100%	2.00 × 10^−71^	99.35%	+/−	CAVMBP010000854
		Last hit	85.1	70%	5.00 × 10^−15^	79.59	+/+	CAVMBP010000752
*P. vulgata*	624	1st hit	166	75%	2.00 × 10^−39^	88.49%	+/−	CAKNZQ020000656
		Last hit	88.7	75%	1.00 × 10^−15^	74.50%	+/−	CAKNZQ020001418
*L. scabra*	111	1st hit	126	89%	2.00 × 10^−27^	85.59%	+/−	JARJEJ010000003
		Last hit	94.2	78%	1.00 × 10^−17^	81.90%	+/+	JARJEJ010000118
*L. gigantea*	113	1st hit	113	70%	4.00 × 10^−24^	81.89%	+/+	AMQO01003418
		Last hit	63.5	78%	6.00 × 10^−9^	71.33%	+/−	AMQO01000500
*N. schrenckii*	16	1st hit	129	86%	7.00 × 10^−29^	86.84%	+/−	JAUJPP010000003
		Last hit	68.0	72%	2.00 × 10^−10^	78.72%	+/−	JAUJPP010000958

## Data Availability

No new data were created or analysed in this study. Data sharing is not applicable to this article.
